# Solitary and periodic wave solutions to the family of new 3D fractional WBBM equations in mathematical physics

**DOI:** 10.1016/j.heliyon.2021.e07483

**Published:** 2021-07-07

**Authors:** Abdulla - Al Mamun, Nur Hasan Mahmud Shahen, Samsun Nahar Ananna, Md. Asaduzzaman, Foyjonnesa

**Affiliations:** a Department of Mathematics, College of Science, Hohai University, Nanjing-210098, PR China; b Department of Mathematics, Islamic University, Kushtia-7003, Bangladesh; c Department of Mathematics, European University of Bangladesh, Dhaka-1216, Bangladesh

**Keywords:** $\left(G^{\prime }/G^{2}\right)$-expansion method, Wazwaz-Benjamin-Bona-Mahony equation, Conformable derivative, Exact solution, Shallow water wave

## Abstract

For the newly implemented 3D fractional Wazwaz-Benjamin-Bona-Mahony (WBBM) equation family, the present study explores the exact singular, solitary, and periodic singular wave solutions via the ($G^{\prime }/G^{2}$)-expansion process. In the sense of conformable derivatives, the equations considered are translated into ordinary differential equations. In spite with many trigonometric, complex hyperbolic, and rational functions, some fresh exact singular, solitary, and periodic wave solutions to the deliberated equations in fractional systems are attained by the implementation of the ($G^{\prime }/G^{2}$)-expansion technique through the computational software Mathematica. The unique solutions derived by the process defined are articulated with the arrangement of the functions tanh, sech; tan, sec; coth, cosech, and cot, cosec. With three-dimensional (3D), two dimensional (2D) and contour graphics, some of the latest solutions created have been envisaged, selecting appropriate arbitrary constraints to illustrate their physical representation. The outcomes were obtained to determine the power of the completed technique to calculate the exact solutions of the equations of the WBBM that can be used to apply the nonlinear water model in the ocean and coastal engineering. All the solutions given have been certified by replacing their corresponding equations with the computational software Mathematica.

## 1 Introduction

Consider the succeeding fractional kind of the WBBM equations [1]:

(1) $$D_{t}^{\gamma }u+D_{x}^{\gamma }u+D_{y}^{\gamma }u^{3}-D_{\mathrm{xzt}}^{3\gamma }u=0,$$

(2) $$D_{t}^{\gamma }u+D_{z}^{\gamma }u+D_{x}^{\gamma }u^{3}-D_{\mathrm{xyt}}^{3\gamma }u=0,$$

(3) $$D_{t}^{\gamma }u+D_{y}^{\gamma }u+D_{z}^{\gamma }u^{3}-D_{\mathrm{xxt}}^{3\gamma }u=0,$$

u(x,y,z,t) is a differentiable function in the above equations with four independent variables x, y, z, and t, and $D_{x}^{\gamma }u$, $D_{y}^{\gamma }u$, $D_{z}^{\gamma }u$, and $D_{t}^{\gamma }u$ denote the corresponding u derivatives of order *γ* with respect to x, y, z, and t respectively, where 0<γ≤1,t≥0. Seadawy et al. [1] explained the equations mentioned above and investigated the variety of soliton solutions. The WBBM equation is defined to describe some particular undular bore formation by a long wave in shallow water [2]. The WBBM equation's derivation dates back to the wave phenomena in the water and the ion-acoustic waves occurring in plasma physics [3]. An analytical solution for an initial boundary value problem with some particular complementary data is suggested by Benjamin et al. [4]. The Lagrangian density and the interaction of two solitary waves for the BBM equation are developed by Morrison et al. [5]. The symmetric WBBM equation is defined by Seyler and Fenstermacher [6] to describe ion-acoustic and space charge waves in the weakly nonlinear sense. Hyperbolic secant type solitary wave solutions and several invariants are also reported in the same work.

It is noteworthy that exact nonlinear PDE solutions are of great importance in explaining various new complex characteristics in various branches of applied science. Different symbolic computational sets, namely Mathematica, Maple, and MATLAB, make it far simpler for physicists, mathematicians, and engineers to build a forum to develop various numerical and analytical methods range of new precise nonlinear PDE solutions. The methods of numeral evolution are the first integral technique [7], the modified Kudryashov technique [8], [9], [10], the modified extended tanh-function method [11], [12], the improved simple equation technique [13], the method of characteristics [14], the novel exponential rational function technique [15], the semi-inverse variational principle [16], [17], the multiple Exp-function system [18], [19], the sine-cosine method [20], the Exp-function method [17], [21], the improved tan⁡(ϕ(ξ)/2) and tanh⁡(ϕ(ξ)/2)-expansion methods [22], [23], the modified trial equation method [24], the extended rational trigonometric method [25], the unified method [26], the Darboux transform method [27], the Adomian decomposition method [28], the exponential rational function method [29], the Bäcklund transformation and inverse scattering method [30], Hirota's bilinear method [31], the advanced exp(−ϕ(ξ))-expansion methods [32], [33], the extended simple equation method [32], the extended sinh-Gordon expansion method [34], [35], [36], [37], the sine-Gordon expansion method [38], [39], [40], the improved ($G^{\prime }/G$) and ($1/G^{\prime }$)-expansion methods [41], the ($G^{\prime }/G$)-expansion method [42], [43], the ($G^{\prime }/G^{2}$)-expansion method [44], [45], the ($G^{\prime }/G$, 1/G)-expansion method [46], [47], [48], variational iteration method [49], [50], [51], the new extended FAN sub-equation method [52], the $\Phi^{6}$-model expansion method [53], the generalized exponential rational function method [54], etc. To date, there has been no credible proof to examine more equations (1)–(3) to check for exact solutions through the ($G^{\prime }/G$, 1/G)-expansion process, which is an expanded version of the simple ($G^{\prime }/G$)-expansion method [42], as far as the authors' awareness is concerned. Quite many researchers have subsequently used the ($G^{\prime }/G$, 1/G)-expansion approach [41], [47], [48] to overcome nonlinear PDEs in diverse fields of use.

This research aims to generate precise solitary wave solutions expending the ($G^{\prime }/G^{2}$)-expansion technique for a deeper appreciation of the physical significance of a diversity of WBBM equations. The arrangement of the tanh, sech; tan, sec; coth, cosech, and cot, cosec functions, expresses the unique solutions excluded by the ($G^{\prime }/G^{2}$)-expansion process. The joint solutions created specify solitary wave, singular periodic, and singular joint solutions.

Among these mentioned approaches, the new investigative process ($G^{\prime }/G^{2}$)-expansion method has been utilized to build exact and explicit solution of time and space-time fractional differential equations. The ($G^{\prime }/G^{2}$) is a useful technique for conniving the traveling wave solutions of nonlinear partial single, coupled and system of equations arising in several expanses of fluid mechanics, physics, water wave mechanics, wave propagation problems, etc. The ($G^{\prime }/G^{2}$)-expansion technique has got much significance due to its general thought and appropriateness. It can be related to numerous nonlinear equations and gives two or three new solutions [47]. The ($G^{\prime }/G^{2}$)-expansion technique is the more efficient and reliable technique as compared to than ($G^{\prime }/G$)-expansion technique. The solutions gained using the mentioned technique can be articulated in trigonometric, hyperbolic, and rational functions. These forms of solutions are satisfactory for reviewing certain nonlinear physical treatment.

In comparison with the attained solutions [47], [48], to the best of our knowledge, kink, bright kink, singular kink, periodic kink, bright and dark bell solution shapes are new in the case of our ($G^{\prime }/G^{2}$)-expansion scheme, which are not testified in previously published studies [41], [47], [48]. It is important to know that most of the investigated solutions in this article have diverse structures over the available solutions in the wave propagation literature. The executed methods are completely new for this studied WBBM equation. Therefore, the developed exact solutions may illuminate the authors for advance studies to clarify pragmatic phenomena in shallow water wave and mathematical physics. This article affords evidence that our mentioned MKE equation is suitable in the sense of conformable derivative for obtaining the new traveling soliton structures in any physical system without any complexity of obliqueness conditions.

The remainder of the paper is decorated as follows: Section 2, the conformable differential equation narration. The $\left(\frac{G^{\prime }}{G^{2}}\right)$-expansion approach has been explained in section 3. We extend this suggested scheme to the 3D fractional WBBM equations in section 4. Physical descriptions and conclusions are collected in sections 5, and 6, respectively.

## 2 Conformable derivative

In this section, we give a brief discussion on conformable derivative, and its properties which follow from the monographs of Khalil et al., [55], Atangana et al., [56] and Abdeljawad [57].

Definition 1 [55] Based on the independent variable *t*, the conformable derivative of order *γ* is defined as

(4) $$D_{t}^{\gamma }\left(z\left(t\right)\right)=\lim_{\rho \to 0}\frac{z\left(t+\rho t^{1-\gamma }\right)-z(t)}{\rho },\;t>0,\;\gamma \in \left(0,1\right],$$

for a persistence z=z(t):[0,∞)→R. This well-defined fractional derivative is achieved by satisfying some known conditions that are necessary.

Theorem 1 *[56]* *Consider the derivative order* γ∈(0,1]*, and assume that for all positive values of t,* g=g(t) *and* f=f(t) *are γ-differentiable. Then,*

• $D_{t}^{\gamma }\left(c_{1}g+c_{2}f\right)=c_{1}D_{t}^{\gamma }\left(g\right)+c_{2}D_{t}^{\gamma }(f)$*.*
• $D_{t}^{\gamma }\left(t^{p}\right)=pt^{p-\gamma },\;\forall p\in R$*.*
• $D_{t}^{\gamma }\left(\mu \right)=0$*,* ∀u(t)=μ*.*
• $D_{t}^{\gamma }\left(\mathrm{gf}\right)=gD_{t}^{\gamma }\left(f\right)+fD_{t}^{\gamma }\left(g\right)$*.*
• $D_{t}^{\gamma }\left(\frac{g}{f}\right)=\frac{fD_{t}^{\gamma }\left(g\right)-gD_{t}^{\gamma }\left(f\right)}{f^{2}}$*.*
• $D_{t}^{\gamma }\left(g\right)\left(t\right)=t^{1-\gamma }\frac{\mathrm{dg}}{\mathrm{dt}}$*,*

*for all* $c_{1},c_{2}\in R$*. Conformable differential operator obeys some crucial essential stuff similar to the chain rule, Taylor series expansion, and Laplace transforms* *[57]**.*

Theorem 2 *Assume* g=g(t) *be a γ conformable differentiable function and assume that* f *is differentiable and well-defined in various* g*. Then,*

*Assume* g=g(t) *is a differentiable function conforming to γ and assume that* f *is differentiable and very well-defined in various g. Then,*

(5) $$D_{t}^{\gamma }\left(g\circ f\right)\left(t\right)=t^{1-\gamma }f^{\prime }\left(t\right)g^{\prime }\left(f\left(t\right)\right),$$

In this study, we have considered the preferred equation with the sense of conformable derivative. In general calculus, several functions do not have Taylor power sequence representations about particular points, but in conformable fractional models, they do have. The conformable derivative performs well in the chain rule and product rule while detailed plans seem normal fractional calculus. The conformable fractional derivative of a constant function is equivalent to zero where it is not the issue for Riemann fractional calculus. Mittag-Leffler functions play a significant role in fractional calculus as a simplification to exponential functions. In contrast, the fractional exponential function $f\left(t\right)=e^{\frac{t^{\alpha }}{\alpha }}$ appears in the case of conformable fractional calculus. Conformable chain rule, conformable fractional derivatives, conformable Gronwall's inequality, conformable integration by parts, conformable Laplace transform, conformable exponential function, and so on, all tend to the corresponding ones in usual calculus [55].

## 3 Description of the $\left(\frac{G^{\prime }}{G^{2}}\right)$-expansion method

In this part, the $\left(\frac{G^{\prime }}{G^{2}}\right)$-expansion method [46] is discussed and evaluated using the suggested methodology.

Consider a nonlinear FDE assumed by

(6) $$F\left(u,D_{t}^{\gamma }u,D_{x}^{\gamma }u,D_{y}^{\gamma }u,D_{t}^{\gamma }D_{t}^{\gamma }u,D_{t}^{\gamma }D_{x}^{\gamma }u,D_{x}^{\gamma }D_{x}^{\gamma }u\right)=0,\;0<\gamma <1.$$

In the above FDE, u(x,y,t) is a mysterious function, and F is a polynomial of u and its partial fractional derivatives.

By expending the complex fractional transformation, we obtain

(7) $$u\left(x,t\right)=U\left(\psi \right),\psi =p\frac{x^{\gamma }}{\gamma }+q\frac{y^{\gamma }}{\gamma }+r\frac{z^{\gamma }}{\gamma }-s\frac{t^{\gamma }}{\gamma },$$

where *p*, *q*, *r*, and *s* are nonzero constants. Equation (7) can be transformed into an ODE of the form:

(8) $$Q\left(U,U^{\prime },U^{\prime \prime },U^{\prime \prime \prime },\ldots \ldots \right)=0.$$

The formula solution of ODE can be written as follows:

(9) $$U\left(\psi \right)=A_{0}+\sum_{k=1}^{N}\left[A_{k}{\left(\frac{G^{\prime }}{G^{2}}\right)}^{k}+B_{k}{\left(\frac{G^{\prime }}{G^{2}}\right)}^{-k}\right],$$

(10) $${\left(\frac{G^{\prime }}{G^{2}}\right)}^{\prime }=\mu +\lambda {\left(\frac{G^{\prime }}{G^{2}}\right)}^{2}.$$

In the above equations, λ≠0 are integers, and $A_{0},\;A_{k},\;B_{k},\;(k=1,2,3,\ldots \ldots ,N)$ are constants to be strong-minded. The value of positive integer N is easy to find by matching the maximum order derivative and nonlinear relations seeming in equation (8).

Substitute equation (9) and use equation (10) into equation (8), accumulate the coefficients with a similar order of ${\left(\frac{G^{\prime }}{G^{2}}\right)}^{j},\;j=0,\pm 1,\pm 2,\ldots \ldots$ and set the coefficients to zero, non-linear algebraic equations are developed. Solutions to the ensuing algebraic system are imitative by using the $\left(\frac{G^{\prime }}{G^{2}}\right)$-expansion technique with the assistance of Mathematica.

Based on the general solutions to equation (10), the ratio $\left(\frac{G^{\prime }}{G^{2}}\right)$ can be separated into three cases as follows:

**Case-I.** Hyperbolic function solution, when (μλ<0)

(11) $$\frac{G^{\prime }}{G^{2}}=-\frac{\sqrt{\left|\mu \lambda \right|}}{\lambda }\left[\frac{a\sinh\left(2\sqrt{\mu \lambda }\psi \right)+a\cosh\left(2\sqrt{\mu \lambda }\psi \right)+b}{a\sinh\left(2\sqrt{\mu \lambda }\psi \right)+a\cosh\left(2\sqrt{\mu \lambda }\psi \right)-b}\right],$$

**Case-II.** Rational function solution, when (μλ=0)

(12) $$\frac{G^{\prime }}{G^{2}}=-\frac{a}{\lambda \left(a\psi +b\right)},\;\mu =0,\;\lambda \neq 0,$$

**Case-III.** Trigonometric function solution, when (μλ>0)

(13) $$\frac{G^{\prime }}{G^{2}}=\sqrt{\frac{\mu }{\lambda }}\left[\frac{a\cos\left(\sqrt{\mu \lambda }\psi \right)+b\sin\left(\sqrt{\mu \lambda }\psi \right)}{b\cos\left(\sqrt{\mu \lambda }\psi \right)-a\sin\left(\sqrt{\mu \lambda }\psi \right)}\right].$$

In the overhead expressions, a and b are nonzero constants.

## 4 Applications of the $\left(\frac{G^{\prime }}{G^{2}}\right)$-expansion method

Here in this sector, we build explicit hyperbolic and periodic solutions for the 3D fractional WBBM equations.

### 4.1 The first 3D fractional WBBM equation

Let the 3D fractional WBBM equation as follows:

(14) $$D_{t}^{\gamma }u+D_{x}^{\gamma }u+D_{y}^{\gamma }u-D_{\mathrm{xzt}}^{3\gamma }u=0.$$

Applying the following wave transformation

$$u\left(x,t\right)=U\left(\psi \right),\;\text{where}\;\psi =p\frac{x^{\gamma }}{\gamma }+q\frac{y^{\gamma }}{\gamma }+r\frac{z^{\gamma }}{\gamma }-s\frac{t^{\gamma }}{\gamma },$$

on equation (14), we get

(15) $$\left(-s+p\right)U^{\prime }+q{\left(U^{3}\right)}^{\prime }+{\mathrm{prsU}}^{\prime \prime \prime }=0.$$

Integrating equation (15) with respect to *ψ*, we get $\left(-s+p\right)U+{\mathrm{qU}}^{3}+{\mathrm{prsU}}^{\prime \prime }+c_{1}=0$, where $c_{1}$ is an integrating constant. We set $c_{1}=0$ for simplicity we get,

(16) $$\left(-s+p\right)U+{\mathrm{qU}}^{3}+{\mathrm{prsU}}^{\prime \prime }=0,$$

with the result of homogeneous balance in equation (16) of the upper order derivative term $U^{\prime \prime }$ and the nonlinear term $U^{3}$, we notice that N=1. Our proposed approach therefore enables us to use the auxiliary solution of the form:

(17) $$U\left(\psi \right)=A_{0}+A_{1}\left(\frac{G^{\prime }}{G^{2}}\right)+B_{1}{\left(\frac{G^{\prime }}{G^{2}}\right)}^{-1}.$$

Now placing the value of U, $U^{\prime \prime }$ and $U^{3}$ in equation (16) and equating the coefficients of similar power of $\left(\frac{G^{\prime }}{G^{2}}\right)$ to zero from the overhead equation we acquire the SAE as follows:

(18) $$\begin{array}{c} pA_{0}-sA_{0}+qA_{0}^{3}+6qA_{0}A_{1}B_{1}=0,\\ pA_{1}-sA_{1}+2prs\lambda \mu A_{1}+3qA_{0}^{2}A_{1}+3qA_{1}^{2}B_{1}=0,\\ pB_{1}-sB_{1}+2prs\lambda \mu B_{1}+3qA_{0}^{2}B_{1}+3qA_{1}B_{1}^{2}=0,\\ 3qA_{0}A_{1}^{2}=0,\\ 3qA_{0}B_{1}^{2}=0,\\ 2prs\lambda^{2}A_{1}+qA_{1}^{3}=0,\\ 2prs\mu^{2}B_{1}+qB_{1}^{3}=0. \end{array}$$

Solving the SAE (18) for $r,\;A_{0},A_{1},B_{1}$ we get some solution sets as follows:

$$r=\frac{p-s}{4ps\lambda \mu },\;A_{0}=0,\;A_{1}=\pm \sqrt{\frac{\left(s-p\right)\lambda }{2q\mu }},\;B_{1}=\pm \sqrt{\frac{\left(-p+s\right)\mu }{2q\lambda }}.r=\frac{-p+s}{8ps\lambda \mu },\;A_{0}=0,\;A_{1}=\pm \frac{1}{2}\sqrt{\frac{\left(p-s\right)\lambda }{2q\mu }},\;B_{1}=\mp \frac{1}{2}\sqrt{\frac{\left(p-s\right)\mu }{q\lambda }}.r=\frac{-p+s}{2ps\lambda \mu },\;A_{0}=0,\;A_{1}=\pm \sqrt{\frac{\left(p-s\right)\lambda }{q\mu }},B_{1}=0.r=\frac{-p+s}{2ps\lambda \mu },\;A_{0}=0,\;A_{1}=0,\;B_{1}=\pm \sqrt{\frac{\left(p-s\right)\mu }{q\lambda }}.$$

Using these solution sets, we construct the solutions to equation (14) as follows:

**When** μλ>0, **we obtain the following trigonometric function solution:**

$$U_{1,2}(x,t)=\pm \frac{(a^{2}+b^{2})\sqrt{\frac{-p+s}{2q}}}{\left(\mathrm{bCos}\left[\delta \right]-\mathrm{aSin}\left[\delta \right]\right)\left(\mathrm{aCos}\left[\delta \right]+\mathrm{bSin}\left[\delta \right]\right)},\delta =\frac{\left(-st^{\gamma }+px^{\gamma }\right)\sqrt{\lambda \mu }}{\gamma }.U_{3,4}(x,t)=\pm \sqrt{\frac{p-s}{q}}\times \frac{\left(-a^{2}+b^{2}\right)\mathrm{Cos}\left[-2\delta \right]+2\mathrm{abSin}\left[-2\delta \right]}{2\left(\mathrm{bCos}\left[\delta \right]-\mathrm{aSin}\left[\delta \right]\right)\left(\mathrm{aCos}\left[\delta \right]+\mathrm{bSin}\left[\delta \right]\right)},\delta =\frac{\left(-st^{\gamma }+px^{\gamma }\right)\sqrt{\lambda \mu }}{\gamma }.U_{5,6}(x,t)=\pm \sqrt{\frac{p-s}{q}}\times \frac{a\;\mathrm{Cos}\left[\delta \right]+b\;\mathrm{Sin}\left[\delta \right]}{b\;\mathrm{Cos}\left[\delta \right]-a\;\mathrm{Sin}\left[\delta \right]},\delta =\frac{\left(-st^{\gamma }+px^{\gamma }\right)\sqrt{\lambda \mu }}{\gamma }.U_{7,8}(x,t)=\pm \sqrt{\frac{p-s}{q}}\times \frac{a\;\mathrm{Sin}\left[\delta \right]-b\;\mathrm{Cos}\left[\delta \right]}{a\;\mathrm{Cos}\left[\delta \right]+b\;\mathrm{Sin}\left[\delta \right]},\delta =\frac{\left(-st^{\gamma }+px^{\gamma }\right)\sqrt{\lambda \mu }}{\gamma }.$$

**When** μλ<0, **we obtain the following hyperbolic function solutions:**

$$U_{9,10}(x,t)=\pm \sqrt{\frac{\left(-p+s\right)\lambda }{2q\mu \left|\lambda \mu \right|}}\;\;\times \frac{-\frac{\left|\lambda \mu \right|}{\lambda }{\left(b+\mathrm{aCosh}\left[\delta \right]-\mathrm{aSinh}\left[\delta \right]\right)}^{2}-\mu {\left(b-\mathrm{aCosh}\left[\delta \right]+\mathrm{aSinh}\left[\delta \right]\right)}^{2}}{\left(b+\mathrm{aCosh}\left[\delta \right]-\mathrm{aSinh}\left[\delta \right]\right)\left(b-\mathrm{aCosh}\left[\delta \right]+\mathrm{aSinh}\left[\delta \right]\right)},\delta =\frac{2\left(st^{\gamma }-px^{\gamma }\right)\sqrt{\left|\lambda \mu \right|}}{\gamma }.U_{11,12}(x,t)=\pm \sqrt{\frac{p-s}{q\lambda \mu \left|\lambda \mu \right|}}\;\;\times \begin{array}{c} \frac{\lambda \mu {\left(\left(a-b\right)\mathrm{Cosh}\left[\delta \right]-\left(a+b\right)\mathrm{Sinh}\left[\delta \right]\right)}^{2}-\left|\lambda \mu \right|{\left(\left(a+b\right)\mathrm{Cosh}\left[\delta \right]+\left(b-a\right)\mathrm{Sinh}\left[\delta \right]\right)}^{2}}{2\left(b+\mathrm{aCosh}\left[2\delta \right]-\mathrm{aSinh}\left[2\delta \right]\right)\left(b-\mathrm{aCosh}\left[2\delta \right]+\mathrm{aSinh}\left[2\delta \right]\right)} \end{array}\;\;\times \left(\mathrm{Cosh}\left[2\delta \right]-\mathrm{Sinh}\left[2\delta \right]\right),\delta =\frac{\left(st^{\gamma }-px^{\gamma }\right)\sqrt{\left|\lambda \mu \right|}}{\gamma }.U_{13,14}(x,t)=\pm \sqrt{\frac{\left(p-s\right)\left|\lambda \mu \right|}{q\lambda \mu }}\times \frac{b+\mathrm{aCosh}\left[\delta \right]-\mathrm{aSinh}\left[\delta \right]}{b-\mathrm{aCosh}\left[\delta \right]+\mathrm{aSinh}\left[\delta \right]},\delta =\frac{2\left(st^{\gamma }-px^{\gamma }\right)\sqrt{\left|\lambda \mu \right|}}{\gamma }.U_{15,16}(x,t)=\pm \sqrt{\frac{\lambda \mu \left(p-s\right)}{q\left|\lambda \mu \right|}}\times \frac{b-\mathrm{aCosh}\left[\delta \right]+\mathrm{aSinh}\left[\delta \right]}{b+\mathrm{aCosh}\left[\delta \right]-\mathrm{aSinh}\left[\delta \right]},\delta =\frac{2\left(st^{\gamma }-px^{\gamma }\right)\sqrt{\left|\lambda \mu \right|}}{\gamma }.$$

### 4.2 The second 3D fractional WBBM equation

Let the 3D fractional WBBM equation as follows:

(19) $$D_{t}^{\gamma }u+D_{z}^{\gamma }u+D_{x}^{\gamma }u-D_{\mathrm{xyt}}^{3\gamma }u=0.$$

Proceeding with the above method, we develop the following solutions:

**When** μλ>0, **we obtain the following trigonometric function solution:**

$$U_{17,18}(x,t)=\pm \frac{i\sqrt{2\mathrm{qs}\lambda \mu }\left(\mathrm{aCos}\left[\delta \right]+\mathrm{bSin}\left[\delta \right]\right)}{\mathrm{aCos}\left[\delta \right]-\mathrm{bSin}\left[\delta \right]},\delta =\frac{\left(-st^{\gamma }+px^{\gamma }\right)\sqrt{\lambda \mu }}{\gamma }.U_{19,20}(x,t)=\pm \frac{2i\sqrt{2\mathrm{qs}\lambda \mu }\left(a^{2}\mathrm{Cos}{\left[\delta \right]}^{2}+b^{2}\mathrm{Sin}{\left[\delta \right]}^{2}\right)}{a^{2}\mathrm{Cos}{\left[\delta \right]}^{2}-b^{2}\mathrm{Sin}{\left[\delta \right]}^{2}},\delta =\frac{\left(-st^{\gamma }+px^{\gamma }\right)\sqrt{\lambda \mu }}{\gamma }.U_{21,22}(x,t)=\pm \frac{2\mathrm{ab}i\sqrt{2\mathrm{qs}\lambda \mu }\;\mathrm{Sin}\left[-2\delta \right]}{a^{2}\mathrm{Cos}{\left[\delta \right]}^{2}-b^{2}\mathrm{Sin}{\left[\delta \right]}^{2}},\delta =\frac{\left(-st^{\gamma }+px^{\gamma }\right)\sqrt{\lambda \mu }}{\gamma }.U_{23,24}(x,t)=\pm \frac{i\sqrt{2\mathrm{qs}\lambda \mu }\left(\mathrm{aCos}\left[\delta \right]-\mathrm{bSin}\left[\delta \right]\right)}{\mathrm{aCos}\left[\delta \right]+\mathrm{bSin}\left[\delta \right]},\delta =\frac{\left(-st^{\gamma }+px^{\gamma }\right)\sqrt{\lambda \mu }}{\gamma }.$$

**When** μλ<0, **we obtain the following hyperbolic function solutions:**

$$U_{25,26}(x,t)=\pm \frac{i\sqrt{2\mathrm{qs}\left|\lambda \mu \right|}\left(b+\mathrm{aCosh}\left[\delta \right]-\mathrm{aSinh}\left[\delta \right]\right)}{b-\mathrm{aCosh}\left[\delta \right]+\mathrm{aSinh}\left[\delta \right]},\delta =\begin{array}{c} \frac{2\left(st^{\gamma }-px^{\gamma }\right)\sqrt{\left|\lambda \mu \right|}}{\gamma } \end{array}.U_{27,28}(x,t)\;=\pm \begin{array}{c} \frac{i\sqrt{2\mathrm{qs}}\left(\left|\lambda \mu \right|{\left(b+\mathrm{aCosh}\left[\delta \right]-\mathrm{aSinh}\left[\delta \right]\right)}^{2}+\lambda \mu {\left(b-\mathrm{aCosh}\left[\delta \right]+\mathrm{aSinh}\left[\delta \right]\right)}^{2}\right)}{\sqrt{\left|\lambda \mu \right|}\left(b+\mathrm{aCosh}\left[\delta \right]-\mathrm{aSinh}\left[\delta \right]\right)\left(b-\mathrm{aCosh}\left[\delta \right]+\mathrm{aSinh}\left[\delta \right]\right)} \end{array},\delta =\frac{2\left(st^{\gamma }-px^{\gamma }\right)\sqrt{\left|\lambda \mu \right|}}{\gamma }.U_{29,30}(x,t)\;=\pm \begin{array}{c} \frac{i\sqrt{2\mathrm{qs}}\left(-\left|\lambda \mu \right|{\left(b+\mathrm{aCosh}\left[\delta \right]-\mathrm{aSinh}\left[\delta \right]\right)}^{2}+\lambda \mu {\left(b-\mathrm{aCosh}\left[\delta \right]+\mathrm{aSinh}\left[\delta \right]\right)}^{2}\right)}{\sqrt{\left|\lambda \mu \right|}\left(b+\mathrm{aCosh}\left[\delta \right]-\mathrm{aSinh}\left[\delta \right]\right)\left(b-\mathrm{aCosh}\left[\delta \right]+\mathrm{aSinh}\left[\delta \right]\right)} \end{array},\delta =\frac{2\left(st^{\gamma }-px^{\gamma }\right)\sqrt{\left|\lambda \mu \right|}}{\gamma }.U_{31,32}(x,t)=\pm \frac{i\sqrt{2\mathrm{qs}}\lambda \mu \left(b-\mathrm{aCosh}\left[\delta \right]+\mathrm{aSinh}\left[\delta \right]\right)}{\sqrt{\left|\lambda \mu \right|}\left(b+\mathrm{aCosh}\left[\delta \right]-\mathrm{aSinh}\left[\delta \right]\right)},\delta =\frac{2\left(st^{\gamma }-px^{\gamma }\right)\sqrt{\left|\lambda \mu \right|}}{\gamma }.$$

### 4.3 The 3^rd^ 3D fractional WBBM equation

Let the 3D fractional WBBM equation as follows:

(20) $$D_{t}^{\gamma }u+D_{y}^{\gamma }u+D_{z}^{\gamma }u-D_{\mathrm{xxt}}^{3\gamma }u=0.$$

Proceeding with the above method, we acquire the following solutions:

**When** μλ>0, **we obtain the following trigonometric function solution:**

$$U_{33,34}(x,t)=\pm \frac{i\sqrt{2}p\sqrt{s}\lambda \sqrt{\frac{\mu }{\lambda }}\left(\mathrm{aCos}\left[\delta \right]+\mathrm{bSin}\left[\delta \right]\right)}{\sqrt{r}\left(\mathrm{aCos}\left[\delta \right]-\mathrm{bSin}\left[\delta \right]\right)},\delta =\frac{\left(-st^{\gamma }+px^{\gamma }\right)\sqrt{\lambda \mu }}{\gamma }.U_{35,36}(x,t)=\pm \frac{2pi\sqrt{2s\lambda \mu }\left(a^{2}\mathrm{Cos}{\left[\delta \right]}^{2}+b^{2}\mathrm{Sin}{\left[\delta \right]}^{2}\right)}{\sqrt{r}\left(a^{2}\mathrm{Cos}{\left[\delta \right]}^{2}-b^{2}\mathrm{Sin}{\left[\delta \right]}^{2}\right)},\delta =\frac{\left(-st^{\gamma }+px^{\gamma }\right)\sqrt{\lambda \mu }}{\gamma }.U_{37,38}(x,t)=\pm \frac{2i\mathrm{abp}\sqrt{2s\lambda \mu }\;\mathrm{Sin}\left[-2\delta \right]}{\sqrt{r}\left(a^{2}\mathrm{Cos}{\left[\delta \right]}^{2}-b^{2}\mathrm{Sin}{\left[\delta \right]}^{2}\right)},\delta =\frac{\left(-st^{\gamma }+px^{\gamma }\right)\sqrt{\lambda \mu }}{\gamma }.U_{39,40}(x,t)=\pm \frac{\mathrm{ip}\sqrt{2s\lambda \mu }\left(\mathrm{aCos}\left[\delta \right]-\mathrm{bSin}\left[\delta \right]\right)}{\sqrt{r}\left(\mathrm{aCos}\left[\delta \right]+\mathrm{bSin}\left[\delta \right]\right)},\delta =\frac{\left(-st^{\gamma }+px^{\gamma }\right)\sqrt{\lambda \mu }}{\gamma }.$$

**When** μλ<0, **we obtain the following hyperbolic function solutions:**

$$U_{41,42}(x,t)=\pm \frac{\mathrm{ip}\sqrt{2s\left|\lambda \mu \right|}\left(b+\mathrm{aCosh}\left[\delta \right]-\mathrm{aSinh}\left[\delta \right]\right)}{\sqrt{r}\left(b-\mathrm{aCosh}\left[\delta \right]+\mathrm{aSinh}\left[\delta \right]\right)},\delta =\frac{2\left(st^{\gamma }-px^{\gamma }\right)\sqrt{\left|\lambda \mu \right|}}{\gamma }.U_{43,44}(x,t)\;=\pm \begin{array}{c} \frac{\mathrm{ip}\sqrt{2s}\left(\left|\lambda \mu \right|{\left(b+\mathrm{aCosh}\left[\delta \right]-\mathrm{aSinh}\left[\delta \right]\right)}^{2}+\lambda \mu {\left(b-\mathrm{aCosh}\left[\delta \right]+\mathrm{aSinh}\left[\delta \right]\right)}^{2}\right)}{\sqrt{r\left|\lambda \mu \right|}\left(b+\mathrm{aCosh}\left[\delta \right]-\mathrm{aSinh}\left[\delta \right]\right)\left(b-\mathrm{aCosh}\left[\delta \right]+\mathrm{aSinh}\left[\delta \right]\right)} \end{array},\delta =\frac{2\left(st^{\gamma }-px^{\gamma }\right)\sqrt{\left|\lambda \mu \right|}}{\gamma }.U_{45,46}(x,t)\;=\pm \begin{array}{c} \frac{\mathrm{ip}\sqrt{2s}\left(-\left|\lambda \mu \right|{\left(b+\mathrm{aCosh}\left[\delta \right]-\mathrm{aSinh}\left[\delta \right]\right)}^{2}+\lambda \mu {\left(b-\mathrm{aCosh}\left[\delta \right]+\mathrm{aSinh}\left[\delta \right]\right)}^{2}\right)}{\sqrt{r\left|\lambda \mu \right|}\left(b+\mathrm{aCosh}\left[\delta \right]-\mathrm{aSinh}\left[\delta \right]\right)\left(b-\mathrm{aCosh}\left[\delta \right]+\mathrm{aSinh}\left[\delta \right]\right)} \end{array},\delta =\frac{2\left(st^{\gamma }-px^{\gamma }\right)\sqrt{\left|\lambda \mu \right|}}{\gamma }.U_{47,48}(x,t)=\pm \frac{\mathrm{ip}\lambda \mu \sqrt{2s}\left(b-\mathrm{aCosh}\left[\delta \right]+\mathrm{aSinh}\left[\delta \right]\right)}{\sqrt{r\left|\lambda \mu \right|}\left(b+\mathrm{aCosh}\left[\delta \right]-\mathrm{aSinh}\left[\delta \right]\right)},\delta =\frac{2\left(st^{\gamma }-px^{\gamma }\right)\sqrt{\left|\lambda \mu \right|}}{\gamma }.$$

## 5 Physical explanation

The physical description of the 3D fractional WBBM equations of the established exact moving wave solutions will be considered in this section. In the ‘physical definition’ portion, the three-dimensional 3D surface plots, contour map, and two-dimensional 2D plots of the developed traveling-wave solutions of the latest 3D fractional WBBM equations are addressed. A 3D line plot highlights the amount of variation over a while or compares multiple wave items. Wave points are designed in series utilizing evenly-spaced breaks and associated with a line to highlight the wave points' relations. The 3D elegance is used to add visual importance to the chart. The 2D line plots are used to represents very high and low frequency and amplitude. The plots are constructed with unique values of γϵ(0,1] at various stages of time using MATLAB for U(x,y,z,t). The plots denote many natures, such as the kink solution, the dark kink type solution, the periodic wave solution, the soliton solution, the singular soliton solution, and other forms of the solution generated by the correct physical description by choosing different free parameters.

**Figure 1:**
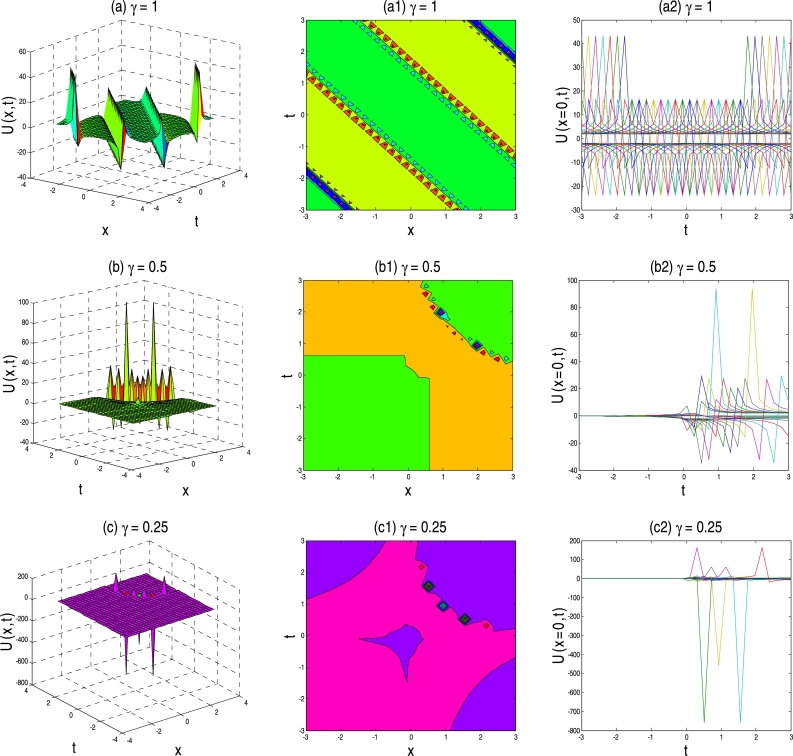
Above set of figures represent the periodic kink solution shape for *γ* = 1, kink shape for *γ* = 0.5 and multiple soliton shape for *γ* = 0.25. Above set of figures belongs to the traveling-wave solution of $U_{1,2}\left(x,t\right)$ for the parameter *a* = 1, *b* = 1, *p* = −0.5, *q* = 0.5, *s* = 0.5, *λ* = 1, *μ* = 1.

**Figure 2:**
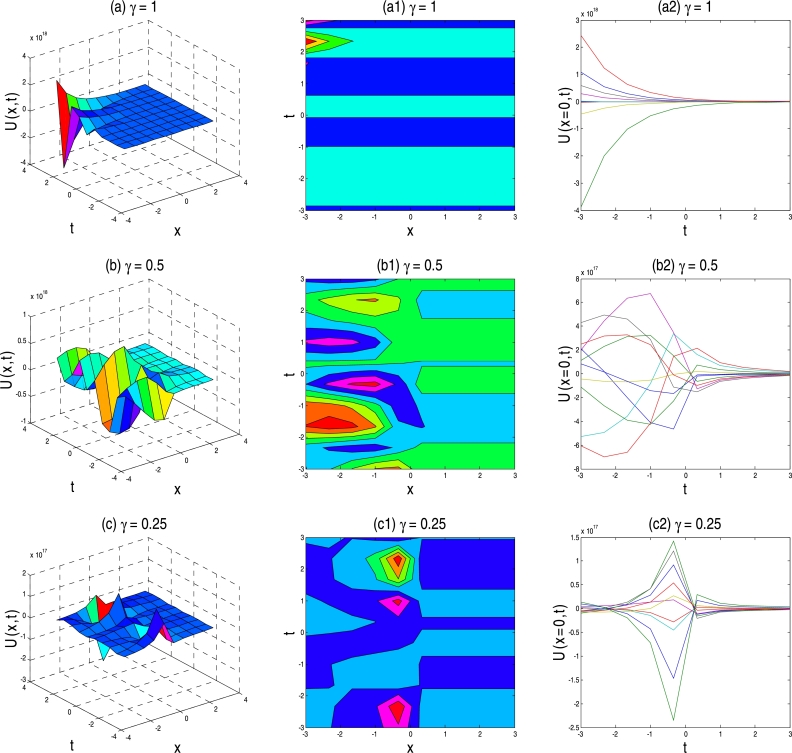
Above set of figures are represents kink solution shape the exact traveling-wave solution of $U_{11,12}\left(x,t\right)$ for the parameter *a* = 1, *b* = 1.5, *p* = −0.5, *q* = 0.5, *s* = 0.5, *λ* = 1, *μ* = −1 and *γ* = 1,0.5,0.25 respectively.

**Figure 3:**
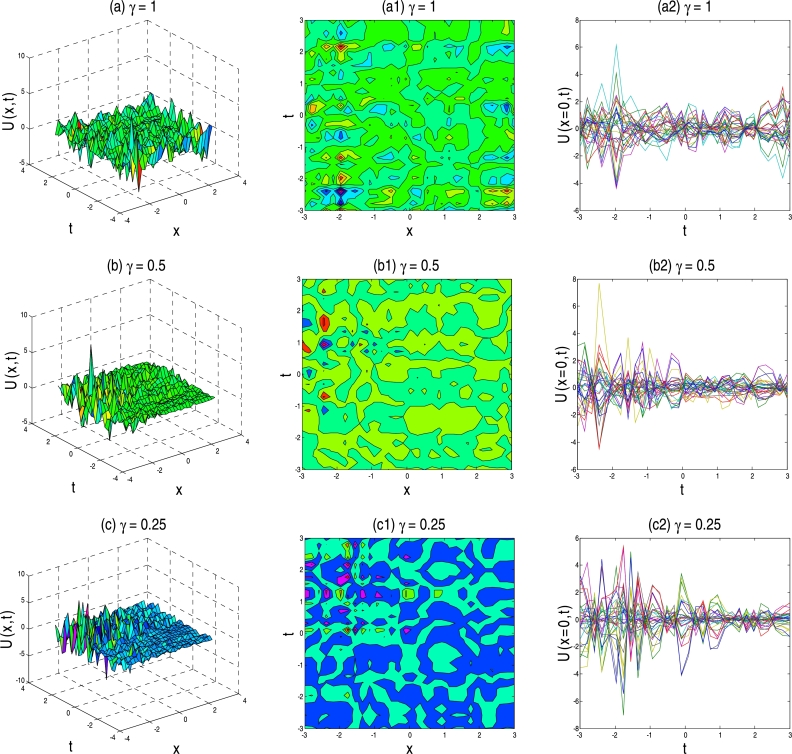
Above set of figures represent the kink solution shape of exact traveling-wave solution of $U_{17,18}\left(x,t\right)$ for the parameter *a* = 1, *b* = 1, *p* = −0.5, *q* = −0.5, *s* = 0.5, *λ* = 1, *μ* = 1 and *γ* = 1,0.5,0.25 respectively.

**Figure 4:**
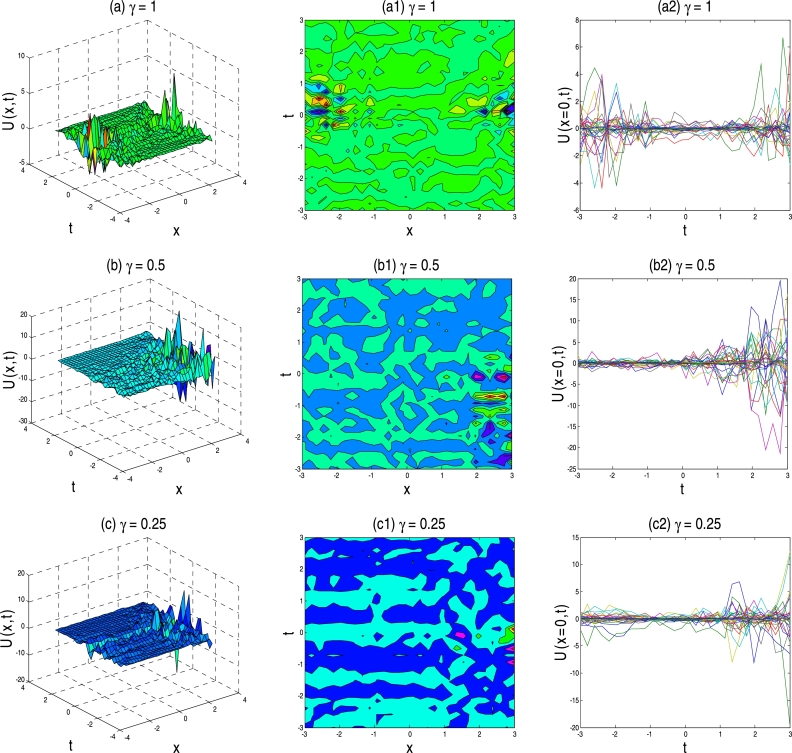
Above set of figures are represents the kink solution shape of exact traveling-wave solution of $U_{31,32}\left(x,t\right)$ for the parameter *a* = 1, *b* = 1.5, *p* = −0.5, *q* = −0.5, *s* = 0.5, *λ* = 1, *μ* = −1 and *γ* = 1,0.5,0.25 respectively.

**Figure 5:**
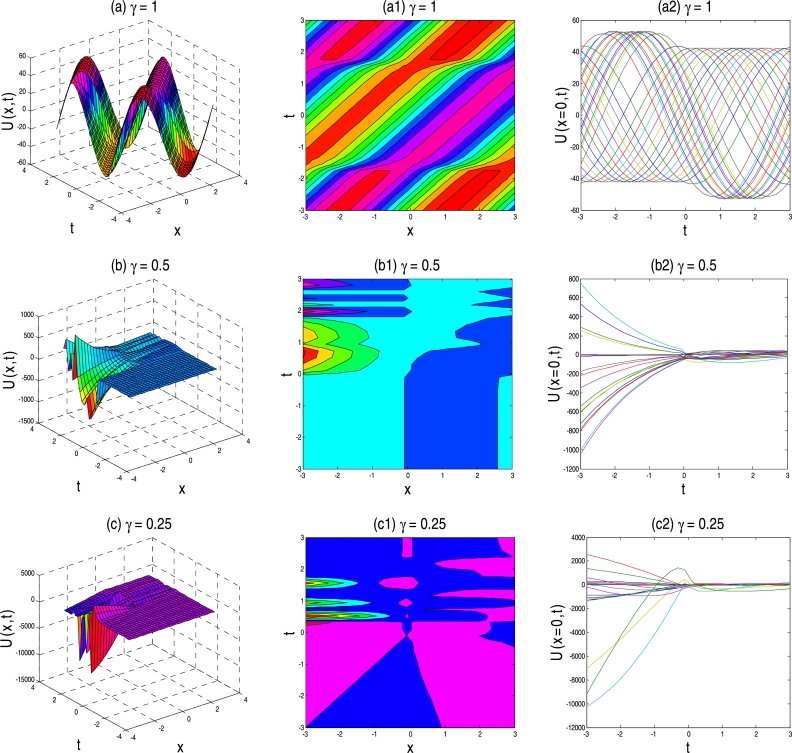
Above set of figures represent the bell solution shape for *γ* = 1, singular kink shape for *γ* = 0.5 and *γ* = 0.25 respectively. Above set of figures belongs to the traveling-wave solution of $U_{37,38}\left(x,t\right)$ for the parameter *a* = 1, *b* = 1, *p* = −0.5, *r* = −0.5, *s* = 0.5, *λ* = 1, *μ* = 1.

**Figure 6:**
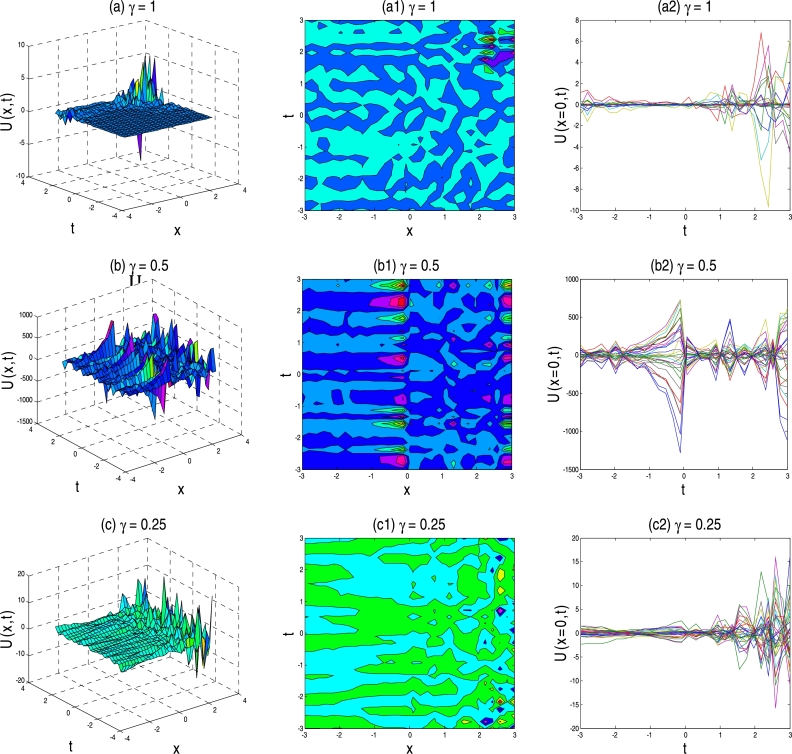
Above set of figures are represents kink solution of the exact traveling-wave solution of $U_{43,44}\left(x,t\right)$ for the parameter *a* = 1, *b* = 1.5, *p* = −0.5, *r* = −0.5, *s* = 0.5, *λ* = 1, *μ* = −1 and *γ* = 1,0.5,0.25 respectively.

In the concept of mathematical physics, a soliton or solitary wave is defined as a self-reinforcing wave packet that upholds its shape. At the same time, it propagates at a constant amplitude and velocity. Soliton is the solutions of a widespread class of weakly nonlinear dispersive partial differential equations describing physical systems. These kink-type solutions' key physical structures are displayed in Figure 1, Figure 2, Figure 3, Figure 4, Figure 5, Figure 6, namely their trajectories, phase shifts after collision and decomposition into separate single kink soliton. With the fractional parameter changes, the wave's frequency and amplitude have been changed, and kink solution shape turned into singular kink. It is necessary to remember that in each set of Figure 1, Figure 2, Figure 3, Figure 4, Figure 5, Figure 6, each appropriate solution has been sketched three times for conformable parameters γ=1, γ=0.5, and γ=0.25 for the first, second and third rows, respectively.

## 6 Conclusion

The $\left(\frac{G^{\prime }}{G^{2}}\right)$-expansion form, exact traveling-wave solutions of the latest 3D fractional WBBM equations are discussed in this article. The equations are simplified to several ODEs at the cost of companionable wave transformation. Then, in the momentous form of ODE, the intended solutions are shared. Some SAE reflects the relation of coefficients of equivalent strength $\left(\frac{G^{\prime }}{G^{2}}\right)$ to zero. Solving this system offers interactions between the parameters. Any physical and hybrid alternatives are specifically unwavering activities of control of a tangent, cotangent, cosecant, hyperbolic tangent, hyperbolic cotangent, and hyperbolic cosecant functions. In specific finite fields, the graphical illustration of some solutions is depicted to explain the results of *γ* by using MATLAB. In this current research, we demand that the solutions obtained are unique and, therefore, more useful in studying the fractional nonlinear dynamics of the water wave and nonlinear mathematical physical phenomena.

### Author contribution statement

Abdulla-Al-Mamun: Conceived and designed the experiments; Performed the experiments; Analyzed and interpreted the data; Contributed reagents, materials, analysis tools or data; Wrote the paper.

Nur Hasan Mahmud Shahen, Md. Asaduzzaman, Foyjonnesa: Analyzed and interpreted the data.

Samsun Nahar Ananna: Performed the experiments.

### Funding statement

This research did not receive any specific grant from funding agencies in the public, commercial, or not-for-profit sectors.

### Data availability statement

No data was used for the research described in the article.

### Declaration of interests statement

The authors declare no conflict of interest.

### Additional information

No additional information is available for this paper.

## References

[1] Seadawy A.R., Ali K.K., Nuruddeen R.I. (2019). A variety of soliton solutions for the fractional Wazwaz-Benjamin-Bona-Mahony equations. Results Phys..

[2] El G.A., Grimshaw R.H.J., Kamchatnov A.M. (2007). Evolution of solitary waves and undular bores in shallow-water flows over a gradual slope with bottom friction. J. Fluid Mech..

[3] Rezazadeh H., Inc M., Baleanu D. (2020). New solitary wave solutions for variants of (3+1)-dimensional Wazwaz-Benjamin-Bona-Mahony equations. Front. Phys..

[4] Benjamin T.B. (1972). The stability of solitary waves. Proc. R. Soc. Lond. Ser. A, Math. Phys. Sci..

[5] Morrison P.J., Meiss J.D., Cary J.R. (1984). Scattering of regularized-long-wave solitary waves. Phys. D: Nonlinear Phenom..

[6] Seyler C.E., Fenstermacher D.L. (1984). A symmetric regularized-long-wave equation. Phys. Fluids.

[7] Eslami M., Mirzazadeh M. (2014). First integral method to look for exact solutions of a variety of Boussinesq-like equations. Ocean Eng..

[8] Kumar D., Darvishi M.T., Joardar A.K. (2018). Modified Kudryashov method and its application to the fractional version of the variety of Boussinesq-like equations in shallow water. Opt. Quantum Electron..

[9] Kumar D., Seadawy A.R., Joardar A.K. (2018). Modified Kudryashov method via new exact solutions for some conformable fractional differential equations arising in mathematical biology. Chin. J. Phys..

[10] Kumar D., Kaplan M. (2018). Application of the modified Kudryashov method to the generalized Schrödinger–Boussinesq equations. Opt. Quantum Electron..

[11] Mamun A.A., An T., Shahen N.H.M., Ananna S.N., Foyjonnesa Hossain M.F., Muazu T. (2020). Exact and explicit travelling-wave solutions to the family of new 3D fractional WBBM equations in mathematical physics. Results Phys..

[12] Alam L.M.B., Jiang X., Mamun A.A. (2021). Exact and explicit travelling traveling wave solution to the time-fractional phi-four and (2+1) dimensional CBS equations using the modified extended tanh-function method in mathematical physics. Part. Diff. Equ. Appl. Math..

[13] Yaşar E., Yıldırım Y., Zhou Q., Moshokoa S.P., Ullah M.Z., Triki H., Biswas A., Belic M. (2017). Perturbed dark and singular optical solitons in polarization preserving fibers by modified simple equation method. Superlattices Microstruct..

[14] Mamun A.-A., Ali M.S., Miah M.M. (2018). A study on an analytic solution 1D heat equation of a parabolic partial differential equation and implement in computer programming. Int. J. Sci. Eng. Res..

[15] Khater M.M.A., Kumar D. (2017). New exact solutions for the time fractional coupled Boussinesq–Burger equation and approximate long water wave equation in shallow water. J. Ocean Eng. Sci..

[16] Darvishi M.T., Najafi M., Wazwaz A.M. (2017). Soliton solutions for Boussinesq-like equations with spatio-temporal dispersion. Ocean Eng..

[17] Liu W. (2009). New solitary wave solution for the Boussinesq wave equation using the semi-inverse method and the Exp-function method. Z. Naturforsch. A.

[18] Ma W.-X., Huang T., Zhang Y. (2010). A multiple exp-function method for nonlinear differential equations and its application. Phys. Scr..

[19] Khatun M.S., Hoque M.F., Rahman M.A. (2017). Multisoliton solutions, completely elastic collisions and non-elastic fusion phenomena of two PDEs. Pramana.

[20] Darvishi M.T., Najafi M., Wazwaz A.M. (2018). Traveling wave solutions for Boussinesq-like equations with spatial and spatial-temporal dispersion. Rom. Rep. Phys..

[21] Rahmatullah Ellahi R., Mohyud-Din S.T., Khan U. (2018). Exact traveling wave solutions of fractional order Boussinesq-like equations by applying Exp-function method. Results Phys..

[22] Manafian J., Lakestani M. (2016). The classification of the single traveling wave solutions to the modified Fornberg–Whitham equation. Int. J. Appl. Comput. Math..

[23] Lakestani M., Manafian J. (2017). Application of the ITEM for the modified dispersive water-wave system. Opt. Quantum Electron..

[24] Odabasi M., Misirli E. (2015). On the solutions of the nonlinear fractional differential equations via the modified trial equation method. Math. Methods Appl. Sci..

[25] Darvishi M.T., Najafi M., Wazwaz A.M. (2018). New extended rational trigonometric methods and applications. Waves Random Complex Media.

[26] Inan B., Osman M.S., Ak T., Baleanu D. (2019). Analytical and numerical solutions of mathematical biology models: the Newell-Whitehead-Segel and Allen-Cahn equations. Math. Methods Appl. Sci..

[27] Li Y., Ma W.-X., Zhang J.E. (2000). Darboux transformations of classical Boussinesq system and its new solutions. Phys. Lett. A.

[28] Zeidan D., Chau C.K., Lu T.-.T., Zheng W.-.Q. (2019). Mathematical studies of the solution of Burgers' equations by Adomian decomposition method. Math. Methods Appl. Sci..

[29] Rezazadeh H., Osman M.S., Eslami M., Mirzazadeh M., Zhou Q., Badri S.A., Korkmaz A. (2019). Hyperbolic rational solutions to a variety of conformable fractional Boussinesq-Like equations. Nonlinear Eng..

[30] Vakhnenko V.O., Parkes E.J., Morrison A.J. (2003). A Bäcklund transformation and the inverse scattering transform method for the generalised Vakhnenko equation. Chaos Solitons Fractals.

[31] Zuo J.-M., Zhang Y.-M. (2011). The Hirota bilinear method for the coupled Burgers equation and the high-order Boussinesq—Burgers equation. Chin. Phys. C.

[32] Shahen N.H.M., Ali M.S., Foyjonnesa, Mamun A.A., Rahman M. (2021). Interaction among lump, periodic, and kink solutions with dynamical analysis to the conformable time-fractional Phi-four equation. Part. Diff. Equ. Appl. Math..

[33] Shahen N.H.M., Bashar M.H., Foyjonnesa, Ali M.S., Mamun A.A. (2020). Dynamical analysis of long-wave phenomena for the nonlinear conformable space-time fractional (2+1)-dimensional AKNS equation in water wave mechanics. Heliyon.

[34] Kumar D., Manafian J., Hawlader F., Ranjbaran A. (2018). New closed form soliton and other solutions of the Kundu–Eckhaus equation via the extended sinh-Gordon equation expansion method. Optik.

[35] Foroutan M., Kumar D., Manafian J., Hoque A. (2018). New explicit soliton and other solutions for the conformable fractional Biswas–Milovic equation with Kerr and parabolic nonlinearity through an integration scheme. Optik.

[36] Seadawy A.R., Kumar D., Chakrabarty A.K. (2018). Dispersive optical soliton solutions for the hyperbolic and cubic-quintic nonlinear Schrödinger equations via the extended sinh-Gordon equation expansion method. Eur. Phys. J. Plus.

[37] Kumar D., Seadawy A.R., Haque M.R. (2018). Multiple soliton solutions of the nonlinear partial differential equations describing the wave propagation in nonlinear low–pass electrical transmission lines. Chaos Solitons Fractals.

[38] Kumar D., Hosseini K., Samadani F. (2017). The sine-Gordon expansion method to look for the traveling wave solutions of the Tzitzéica type equations in nonlinear optics. Optik.

[39] Kumar D., Seadawy A.R., Chowdhury R. (2018). On new complex soliton structures of the nonlinear partial differential equation describing the pulse narrowing nonlinear transmission lines. Opt. Quantum Electron..

[40] Hosseini K., Kumar D., Kaplan M., Bejarbaneh E.Y. (2017). New exact traveling wave solutions of the unstable nonlinear Schrödinger equations. Commun. Theor. Phys..

[41] Khater M.M.A., Kumar D. (2017). Implementation of three reliable methods for finding the exact solutions of (2 + 1) dimensional generalized fractional evolution equations. Opt. Quantum Electron..

[42] Wang M., Li X., Zhang J. (2008). The ($G^{\prime }/G,1/G$)-expansion method and travelling wave solutions of nonlinear evolution equations in mathematical physics. Phys. Lett. A.

[43] Younis M. (2017). Optical solitons in (n + 1) dimensions with Kerr and power law nonlinearities. Mod. Phys. Lett. B.

[44] Bilal M., Seadawy A.R., Younis M., Rizvi S., El-Rashidy K., Mahmoud S.F. (2021). Analytical wave structures in plasma physics modelled by Gilson-Pickering equation by two integration norms. Results Phys..

[45] Bilal M., Seadawy A.R., Younis M., Rizvi S.T.R., Zahed H. (2020). Dispersive of propagation wave solutions to unidirectional shallow water wave Dullin–Gottwald–Holm system and modulation instability analysis. Math. Methods Appl. Sci..

[46] Li L., Li E., Wang M.-. (2010). The (G′/G)-expansion method and its application to travelling wave solutions of the Zakharov equations. Appl. Math. J. Chin. Univ. Ser. A.

[47] Mamun Miah M., Shahadat Ali H.M., Ali Akbar M., Majid Wazwaz A. (2017). Some applications of the (G′/G, 1/G)-expansion method to find new exact solutions of NLEEs. Eur. Phys. J. Plus.

[48] Mamun A.A., Ananna S.N., An T., Shahen N.H.M., Foyjonnesa (2021). Periodic and solitary wave solutions to a family of new 3D fractional WBBM equations using the two-variable method. Part. Diff. Equ. Appl. Math..

[49] Ananna S.N., Mamun A.-A.-. (2020). Solution of Volterra's integro-differential equations by using variational iteration method. Int. J. Sci. Eng. Res..

[50] Mamun A.-A., Asaduzzaman M., Ananna S.N. (2019). Solution of eighth order boundary value problem by using variational iteration method. Int. J. Math. Comput. Sci..

[51] Mamun A.-A., Asaduzzaman M. (2019). Solution of seventh order boundary value problem by using variational iteration method. Int. J. Math. Comput. Sci..

[52] Osman M.S., Tariq K.U., Bekir A., Elmoasry A., Elazab N.S., Younis M., Abdel-Aty M. (2020). Investigation of soliton solutions with different wave structures to the (2 + 1)-dimensional Heisenberg ferromagnetic spin chain equation. Commun. Theor. Phys..

[53] Seadawy A.R., Bilal M., Younis M., Rizvi S., Althobaiti S., Makhlouf M. (2021). Analytical mathematical approaches for the double-chain model of DNA by a novel computational technique. Chaos Solitons Fractals.

[54] Bilal M., Shafqat-ur-Rehman, Younas U., Baskonus H.M., Younis M. (2021). Investigation of shallow water waves and solitary waves to the conformable 3D-WBBM model by an analytical method. Phys. Lett. A.

[55] Khalil R., Al Horani M., Yousef A., Sababheh M. (2014). A new definition of fractional derivative. J. Comput. Appl. Math..

[56] Atangana A., Baleanu D., Alsaedi A. (2015). New properties of conformable derivative. Open Math..

[57] Abdeljawad T. (2015). On conformable fractional calculus. J. Comput. Appl. Math..

